# Targeting autophagy for breast cancer prevention and therapy: From classical methods to phytochemical agents

**DOI:** 10.22038/ijbms.2024.79405.17201

**Published:** 2024

**Authors:** Sadegh Rajabi, Heewa Shakib, Nahid Safari-Alighiarloo, Marc Maresca, Maryam Hamzeloo-Moghadam

**Affiliations:** 1 Traditional Medicine and Materia Medica Research Center, Shahid Beheshti University of Medical Sciences, Tehran, Iran; 2 Cellular and Molecular Endocrine Research Center, Research Institute for Endocrine Sciences, Shahid Beheshti University of Medical Sciences, Tehran, Iran; 3 Endocrine Research Center, Institute of Endocrinology and Metabolism, Iran University of Medical Sciences, Tehran, Iran; 4 Aix Marseille Univ, CNRS, Centrale Marseille, iSm2, 13013 Marseille, France; 5 Traditional Medicine and Materia Medica Research Center and Department of Traditional Pharmacy, School of Traditional Medicine, Shahid Beheshti University of Medical Sciences, Tehran, Iran

**Keywords:** Autophagy, Breast cancer, Cell death, Phytochemicals, Targeted therapy

## Abstract

Breast cancer is a heterogeneous illness comprising diverse biological subtypes, each of which differs in incidence, response to therapies, and prognosis. Despite the presence of novel medications that effectively target vital cellular signaling pathways and their application in clinical practice, breast cancer can still develop resistance to therapies by various mechanisms. Autophagy is a conserved catabolic cellular process that maintains intracellular metabolic homeostasis by removing dysfunctional or unnecessary cellular materials to recycle cytosolic components. This process serves as an adaptive survival response to diverse stress stimuli, thereby contributing to tumor initiation, progression, and drug resistance, leading to restriction of the effectiveness of chemotherapeutic treatments. Regarding this potential role of autophagy, molecular regulation and signal transduction of this process represent an attractive approach to combat cancer development and drug resistance. Among various therapeutic agents, bioactive plant-derived compounds have received significant interest as promising anticancer drugs. A plethora of evidence has shown that phytochemicals with the capacity to modulate autophagy may have the potential to be used as inhibitors of breast cancer growth. In this review, we describe recent findings on autophagy targeting along with conventional methods for breast cancer therapy. Subsequently, we introduce phytochemical compounds with the capacity to modulate autophagy for breast cancer treatment.

## Introduction

Breast cancer is the most common cancer globally, and estimates show that the burden of breast cancer will increase to more than 3 million new cases and 1 million deaths annually by 2040 (1). Breast cancer can be quietly cured using current therapeutic methods, especially when the disease is in its early stages. However, many breast cancer patients resist regular pharmacological therapies (2). Thus, a deeper comprehension of the processes leading to the emergence of resistance contributes to improving current treatment regimens and may result in developing new remedies that help to overcome therapy resistance.

Autophagy, which translates to “self-eating,” is a tightly controlled catabolic cellular activity conserved from yeast to more complex eukaryotes such as humans (3). Cytoplasmic components are transported into the lysosome, where resident hydrolases break them down to maintain cellular homeostasis. The breakdown products are then recycled into the cytoplasm (3). Since basal autophagy inhibits the gradual accumulation of damaged protein aggregates and organelles, it acts as the quality-control machinery for cytoplasmic components and is crucial for homeostasis (4). Additionally, the proper function of autophagy is essential for preserving other aspects of cellular physiology, such as well-organized cell metabolism and genomic integrity (5). 

Various pathological conditions such as cancer, aging, metabolic disorders, and neurological illnesses are associated with autophagy dysfunction (6). The role of autophagy in cancer is more complicated than other pathophysiological conditions due to its dual role in suppressing or promoting carcinogenesis (7). Several factors affect autophagy’s paradoxical function in tumorigenesis, including tumor stage, nutrient availability, immune status, pathogenic conditions, and microenvironment stress (8). Autophagy suppresses tumor development in the early stages. Still, several studies have indicated that autophagy plays an important role in cancer progression in the late stages after establishing cancer (9). Autophagy also has a diverse effect on breast cancer, including tumor-suppressive and tumor-promoting impacts at every stage of tumorigenesis (10).

Plants contain a variety of phytochemicals that are beneficial for human health (11). Phytochemical compounds show many biological activities, including anticancer, anti-inflammatory, anti-oxidant, and antimicrobial effects (12). These compounds show anticancer effects by inducing cell death, targeting signaling pathways, regulating oxidative stress, and inhibiting metastasis in cancer cells (13). Various phytochemicals have been shown to act as potent autophagy modulators in cancer cells (14). This review article overviews the molecular mechanisms implicated in autophagy and the importance of autophagy targeting in breast cancer. Additionally, we outline recent findings on autophagy modulation combined with conventional methods for treating breast cancer. Finally, we introduce phytochemical compounds that modulate autophagy to inhibit breast cancer initiation, promotion, and progression. 

**Figure 1:**
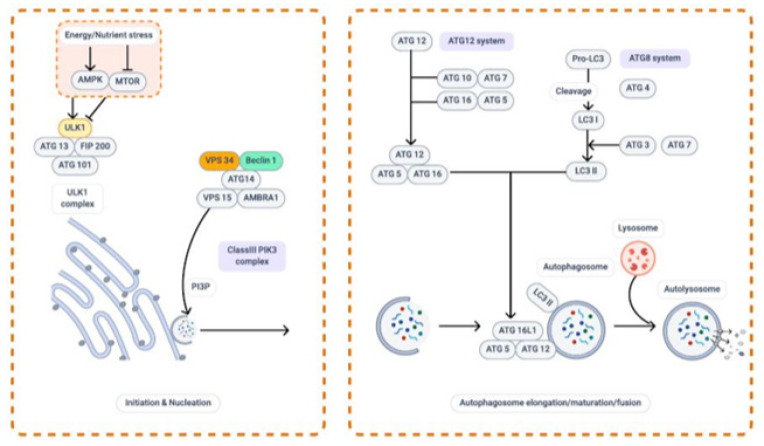
Overview of autophagy process

Initiation: Autophagy is triggered under stresses like starvation, inducing mTORC1 inhibition and releasing ULK1. Consequently, ULK1 phosphorylates and activates Atg13, Atg101, and FIP200. Adenosine monophosphate (AMP)-activated protein kinase (AMPK), which measures the energy status of the cell, also stimulates the ULK complex. Nucleation: ULK1 protein phosphorylates and activates a class III phosphatidylinositol 3-kinase (PI3KC3) complex. This complex consists of Beclin1, Vps34, Vps15, AMBRA1, UVRAG (p63), and Atg14. As a result of the activation of the PI3KC3 complex, PI3P is produced at the site of phagophore formation, which is essential for subsequent autophagy regulatory proteins to be recruited. Elongation/Expansion: The phagophore expands using multiple lipid sources to form enclosed vesicles called autophagosomes upon initiation and nucleation. Autophagosome formation is predominantly modulated by two ubiquitin-like conjugation systems, ATG5-ATG12 and ATG8-phosphatidylethanolamine (PE). These systems involve ATG5, ATG12, ATG10, ATG16, and the ATG8 family members, including LC3 and GABARAP subfamilies, which conjugate to the lipids and recruit to the membrane. Maturation and Fusion: Upon completion, autophagosomes undergo a maturation process that seems to occur almost simultaneously with the separation from the endoplasmic reticulum (ER). Maturation is associated with the dissociation of PIP3 by phosphoinositide phosphatases and the cleavage of Atg8/LC3 through the action of the Atg4 protease family. A mature phagophore eventually surrounds the destined cargo and fuses to form a double membrane phagosome. Once most ATG machinery is released, the encapsulated cargo is transported into the acidic and hydrolytic lysosomal lumen to be degraded. This final step necessitates the fusion of the outer autophagosomal membrane with the lysosome, leading to the formation of the autolysosome. LC3 (Microtubule-associated protein 1A/1B-light chain 3) plays an important role in autophagosome formation, selecting substrates for lysis by the autolysosome. LC3 is synthesized as pro-LC3, processed, and cleaved by ATG4 to produce LC3-I, which is then conjugated to phosphatidylethanolamine (PE) by Atg7 and Atg3 to form LC3-II. LC3-II is located on the outer or inner side of the expanding phagophore's membrane, and its presence is critical for autophagy regulation.

**Table 1:** Relationship between autophagy and classical methods of breast cancer therapy

Therapy method	Therapeutic agent	Adjuvant	Breast cancer type	Autophagy alteration	Cell viability	Ref.
Radiation therapy	Ionizing radiation	Bafilomycin A1, Chloroquine	TNBC	Inhibition	Decrease	53
	Ionizing radiation	Chk1 inhibitor MK-8776	TNBC	Inhibition	Decrease	54
	Ionizing radiation	No	TNBC	Induction	Decrease	55
Chemotherapy	Trastuzumab	Chloroquine	HER2^+^	Inhibition	Decrease	57
	Paclitaxel	Chloroquine	TNBC	Inhibition	Decrease	58
	Docetaxel	ATG7 siRNA	ER^+^/PR^+^	Inhibition	Decrease	59
	Doxorubicin, 5-fluorouracil	Bafilomycin A1, ATG5 siRNA	TNBC	Inhibition	Decrease	60
	Paclitaxel, Doxorubicin, Carboplatin	ADIPOQ/adiponectin	TNBC, ER^+^/PR^+^	Induction	Decrease	62
	Doxorubicin	Estrogen	ER^+^/PR^+^	Induction	Decrease	63
Hormonal therapy	Tamoxifen	siRNA-mediated knockdown of autophagy markers LAMP3, MAP1LC3B, ATG5, and BECN1	ER^+^/PR^+^	Inhibition	Decrease	65
	Tamoxifen	HDACI, Chloroquine, Beclin 1 siRNA	ER^+^/PR^+^	Inhibition	Decrease	64
	Exemestane	No	ER^+^/PR^+^	Induction	Decrease	70
	Lapatinib (Short term treatment)	No	HER2^+^	Induction	Decrease	73
	Lapatinib	G6PD inhibition	ER^+^/PR^+^	Induction	Decrease	74
	Trastuzumab emtansine	No	HER2^+^	Induction	Decrease	76

Chk1: Checkpoint kinase 1; TNBC: Triple-negative breast cancer; ER: Estrogen receptor; PR: Progesterone receptor; HER2: Human epidermal growth factor receptor 2; G6PD: Glucose-6-phosphate dehydrogenase; HDACI: Histone deacetylase inhibitor

**Table 2:** Phytochemicals and their role in modulating autophagy in breast cancer

Phytochemical	Cancer models	Molecular effects	Outcome	Concentration	Ref.
Artemisinin/ Artesunate	MCF-7 MDA-MB-231	Up-regulation of Beclin-1 Up-regulation of P21 Aggregation of LC3II Cell cycle arrest in the G2/M phase	Increased sensitivity to the chemotherapy drug epirubicin	50 µg/ml	[108]
	MDA-MB-231 cisplatin-resistant cancer cells	Promoting autophagosome formation Creates autophagic vacuoles	Triggering apoptosis, autophagy, G2/M phase arrest.	25, 50, 100 µM	[109]
	MCF-7 MDA-MB-231 T47D	Inhibiting autophagosome turnover and causing perinuclear clustering of autophagosomes, endosomes, and lysosomes	Mitochondrial apoptosis Lysosomal cell death	10, 20 µg/ml	[110]
	MDA-MB-231 MCF-7 4T1 Xenograft tumors	Decrease PHB2 Increase LC3-II and PINK1	Inhibition of cell proliferation via mitophagy	2.5, 5, 10, 20 µM (*in vitro*) 15 or 30 mg/kg (*in vivo*)	[111]
Baicalein	MDA-MB-231 MCF-7 Xenograft tumor	Reduced p-AKT, p-mTOR, NF-κB, and p-IκB expression Increased IκB expression Reduced p-AKT/AKT and p-mTOR/mTOR ratios	Suppressing cell proliferation, Triggering apoptosis and autophagy	10-40 µM	[115]
	MDA-MB-231	Decreased autophagy markers upregulated CDK1	Improves the chemosensitivity of TNBC cells to doxorubicin.	20 µM	[116]
	MDA-MB-231	Activated AMPKα and ULK1 Down-regulated mTOR and Raptor	autophagic cell death	5 μg/ml	[117]
Britannin	MCF-7	Down-regulation of ATG1, ATG4, ATG5, Beclin1, and LC-III	Induction of apoptosis and inhibition of autophagy	25 µM	[118]
Celastrol	MCF-7 zebrafish xenograft model	Elevated expression of LC3 A/B, p62 and Beclin-1	Antiproliferative activity Inhibited the colony formation of the MCF-7 cells	1.31 μM	[121]
	MCF-7 MCF-7-implanted nude mouse model	Increase in the autophagy marker proteins LC3II and P62	In combination with tamoxifen exerted synergistic effects	Celastrol (0.3 μM), TAM(1, 3, and 10 μM,)	[122]
Cucurbitacin B	MCF-7	Up-regulated of LC3 II Inhibited protein expressions of p-mTOR, p-Akt, and p62, Enhanced expressions of Beclin-1 and p-ULK1	Decreased cell viability Caused DNA damage Induced autophagy	0-200 nM	[129]
Curcumin	MCF7 xenograft model	Down-regulation of long noncoding RNA Colon-cancer-associated transcript-1 (CCAT1) Inactivation of PI3K/Akt/mTOR pathway	Sensitized MDR breast cancer cells to cisplatin	20 µM	[132]
	MDA-MB-231	Autophagic vacuoles formation	Apoptosis Autophagy Cell cycle arrest	25 μg/mL	[133]
	MDA-MB-231 MCF-7	Formation of autophagic vacuoles, the turnover of LC3-II, and the degradation of p62	Synergistic chemopreventive Effects through induction of apoptosis and autophagic cell death	2.5, 5, 10 µM	[134]
Gaillardin	MCF-7	Down-regulation of ATG1, ATG4, ATG5, Beclin1, and LC-III	Induction of apoptosis and inhibition of autophagy	15 µM	[135]
Icariin	MCF-7	Down-regulated the expression of CDK2, CDK4, Cyclin D1, Bcl-2, LC3-1, LC3-II, AGT5, Beclin-1 Up-regulated the expression of caspase-3, PARP and p62	Reverse resistance to tamoxifen in the TAM-R cells induced cell cycle G0/G1 phase arrest and apoptosis through suppression of autophagy	10-75 μM	[60]
	MDA-MB-231 MCF-7 xenograft model	Increased the number of autophagosomes and LC3 II expression	Cytoprotective autophagy Induced apoptosis Inhibition of tumor growth	20 µM (*in vitro*) 15 mg/kg (*in vivo*)	[139]
Paclitaxel	BT474 xenograft model	Reduced Beclin1 expression	Inhibition of tumor growth	0.5 μM	[140]
Paxitaxel + Silence eEF2K	MDA-MB-231 MDA-MB-468	Decreased LC3 I LC3 II expression	Enhanced autophagy, Decreased growth and invasion of PTX-resistant tumor cells using eEF2K silencing	1-12 nM	[64]
Paxitaxel + spautin-1 or knockdown of FIP200 and Atg13	MDA-MB-231 cells	Accumulation of damaged mitochondria	Enhanced paclitaxel-induced cell death	25 nM	[145]
Paxitaxel + TIPE2	MCF-7 MDA-MB-231	Activating AKT/mTOR Inhibiting TAK1/MAPK Down-regulated Beclin1 and LC3B (LC3II/I)	Sensitized breast cancer cells to PTX	20, 25 nM	[146]
Resveratrol	MDA-MB-231 MCF-7	Suppression of AKT signaling Inhibition of autophagy flux	Sensitized breast cancer cells to talazoparib	Talazoparib (200nM ) Resveratrol (10, 20, 40µM )	[147]
	ER+ and ER- cancer cells	Inhibition of the mTORC1/S6K1 signaling Preventing up-regulation of Akt activation	In combination with rapamycin blocked up-regulation of autophagy and induced apoptosis	Rapamycin (20nM ) Resveratrol (100µM )	[148]
	MDA-MB-231 T47D	Beclin1 up-regulation Induction of autophagy	Initiated tumor cell death	Aza resveratrol analogs (50µM)	[149]
	MCF-7 SUM159 xenograft model	Suppressing the Wnt/b-catenin pathways Increased LC3-II, Beclin1 and Atg 7	Inhibited the growth of xenograft tumors Reduced BCSC population in tumor cells	40 µM (*in vitro*) 100 mg/kg (*in vivo*)	[150]
	4T1 xenograft model	Phosphorylating AMPK Enhancing the expression of SIRT3 Formation of autophagic vacuoles enhanced the expression of Beclin 1 and LC3β	Inhibition of autophagy, proliferation, invasion, metastasis of tumor cells	25 μM	[151]
	MDA-MB-231 Tumorosphere model	Decreased expression of Beclin, LC3I, and LCII Increased Bax/Bcl-2 ratios	Suppress EMT, inflammation, autophagy, and apoptosis of tumor cells	Resveratrol (72 μM) salinomycin (1.34 μM)	[152]
	MCF-7	Beclin 1-independent autophagy Increased LCII	Apoptotic cell death	16, 32, 64 μM	[153]
Tetrandrine	MDA-MB-231	Elevated Beclin1, LC3-II/LC3-I Reduced p62/SQSTM1 PI3K/AKT /mTOR	Blocked cell proliferation and stimulated autophagy and apoptosis	12.8, 16.1, 25.7 *μ*M	[155]
	MCF-7	Induces Autophagy via PKC-α inhibition	Amplified autophagic flux leading to cell death	0-20 µM	[156]
	TAM-R MCF-7	Increased autophagosomes and the levels of LC3-II and p62	Suppressed TAM-resistant cells’ growth Potentiates the pro-apoptotic effect of TAM via inhibition of autophagy	Tet (0.9 1.8 and 3.75 μg/ml) TAM (1 μM)	[157]
	MCF-7	Up-regulated LC3-I, LC3-II, ATG7, Beclin-1, AMPK Down-regulated mTOR	Cell cycle arrest, autophagic cell death	Arsenite 4 μM Tetra 2 μg/ml	[158]
Thymoquinone	MDA-MB-231	Declined Beclin-1 and LC3 expression	Autophagy inhibition Suppressing pathways of cell migration, invasion, angiogenesis	1.6 to 3.125 µM	[160]
	MCF-7 T47D	Increased autophagic vesicles	Suppress cell proliferation and migration Synergizes anti-breast cancer effects of gemcitabine	TQ and GCB (0.01 to 300 µM)	[161]
	MCF-7 T47D	Increased expression of Beclin-1 and LC3-II	Sensitizes breast cancer cells to PTX-killing effects through autophagy	TQ (0.01–300 µM), PTX (0.001–10 µM)	[105]
	MCF-7 MDA-MB-231	Increased autophagic vesicles	synergistic effect on stimulating autophagy and apoptosis	0.01–100 μM	[162]
Tocotrienols	MCF-7 and MDA-MB-231	Increase in autophagy markers of early phase (Beclin-1, LC3B-II) and late phase (LAMP-1 and cathepsin-D)	Decreased cancer cell viability	40 μM	[165,166]
	Malignant +SA mouse mammary epithelial cells	Increase in conversion of LC3B-I to LC3B-II, beclin-1, Atg3, Atg7, Atg5-Atg12, LAMP-1 and cathepsin-D	In combination with oridonin-induced synergistic autophagic and apoptotic effects	γ-tocotrienol (8 µM) and oridonin (2 µM)	[167]
Ursolic acid	MCF-7 (ER+, PR+/−, HER2−) MDA-MB-231 (ER−, PR−, HER2−) SK-BR-3 (ER−, PR−, HER2+)	Diminished AKT signaling Affected glycolysis as judged by decreased levels of HK2, PKM2, ATP, and lactate Activated AMPK due to induction of energy stress	Resulted in cytotoxic autophagy and apoptosis	5–20 µM	[168]
	MCF-7 MDA-MB-231	Raised the expression of Beclin-1, LC3-II/LC3-I, Atg5, and Atg7, and while lessening PI3K and AKT levels	Sensitize breast cancer cells to epirubicin (EPI)	EPI (20- 80 µM) UA (1-20 µM)	[169]
	T47D MCF-7 MDAMB231	Reduced PI3K Reduced AKT phosphorylation Increased LC3 Decreased cyclin‑D1 Increased caspase-3 Down-regulated NF‑κB	Inhibit proliferation, Induce autophagy and apoptosis Suppressing inflammation in tumor cells	160 μg/ml	[170]
	MCF-7	Increased ATG5 and BECN1 Activated MAPK1/3 Increased EIF4EBP1 and RPS6KB1/2 phosphorylation	Autophagy-dependent ER stress protected the tumor cells from UA-induced apoptosis	10-20 μM 25–30 µM	[171]

**Figure 2:**
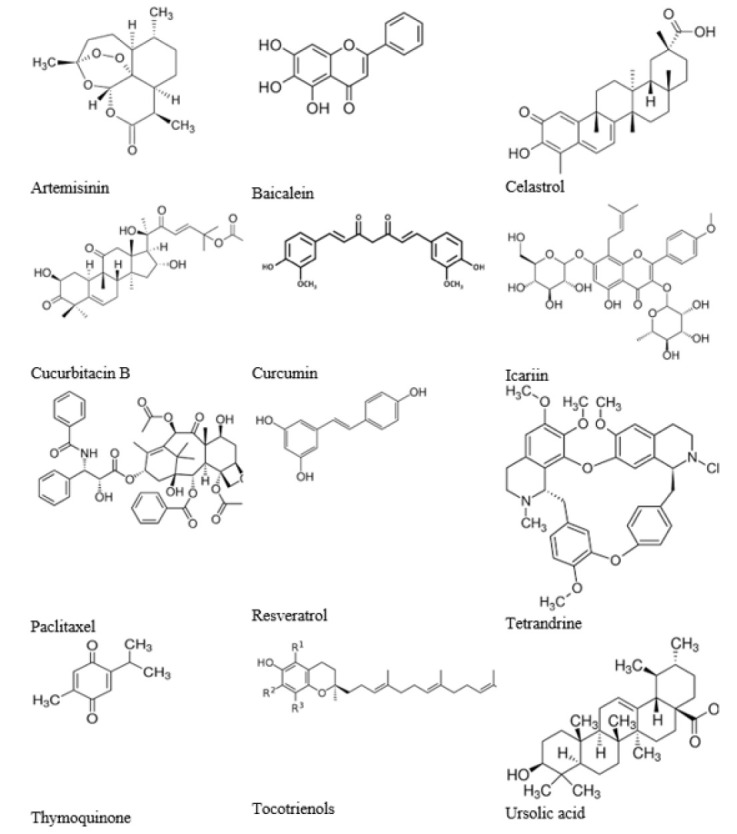
Chemical structure of phytochemicals with capacity to target autophagy in breast cancer

***Molecular mechanisms of autophagy***

There are three different types of autophagy: 1) macroautophagy, which is characterized by the formation of a unique double-membrane organelle of autophagosome; 2) microautophagy, which involves the lysosome membrane invagination, protrusion, and septation to engulf cytoplasm at the lysosome surface directly; and 3) chaperone-mediated autophagy, which is mediated by direct translocation of proteins across the lysosomal surface through a putative pore (15). Macroautophagy is the best-studied type and will be referred to as autophagy in this review. Autophagy can also be categorized into selective and nonselective autophagy based on how cargo is delivered to the lysosome (16). Damaged proteins (aggrephagy), damaged organelles such as mitochondria (mitophagy), endoplasmic reticulum (endoplasmic reticulum-phagy), and invaded pathogens (xenophagy) are degraded by selective autophagy. In contrast, nonselective autophagy is triggered during cellular stress conditions such as starvation, which converts cytoplasmic contents into molecules rich in energy to be used for cell recovery (17).

Autophagy involves the formation of a double-membrane vesicle known as autophagosome, which is encapsulated by autophagy-related gene products (ATGs). By fusing with lysosomes, the autophagosome degrades its contents via lysosomal hydrolase activity (18). An autophagosome is typically formed by passing the following stages: initiation, phagophore nucleation, phagophore membrane elongation/expansion, autophagosome maturation, and fusion with the lysosome. Figure 1 depicts a schematic representation of the autophagy process in intracellular spaces.

***Initiation***

Several factors can activate autophagy, including starvation, hypoxia, deficiency of growth factors, ATP/AMP ratio, intracellular reactive oxygen species (ROS) levels, pathogens, and chemical agents (19). The phagophore assembly site (PAS) is a subcellular location where ATGs are recruited following the activation of intracellular signaling pathways (20). The origin of the phagophore and PAS in mammalian cells is currently a topic of debate. However, increasing evidence shows that the phagophore formation occurs in an endoplasmic reticulum (ER) membrane subdomain called omegasome. This membrane is enriched with phosphatidylinositol 3-phosphate (PI3P) (21).

Autophagosome formation is initiated by the assembly of UNC-51-like kinase (ULK) complex comprising ULK1, ULK2, FIP200, ATG13, and ATG101 (22). Autophagy is suppressed in normal conditions by the mechanistic target of rapamycin complex 1 (mTORC1), which phosphorylates and inhibits the ULK1 complex (23). Autophagy is triggered under stresses like starvation, inducing mTORC1 inhibition and releasing ULK1. Consequently, ULK1 phosphorylates and activates Atg13, Atg101, and FIP200 (24). Adenosine monophosphate (AMP)-activated protein kinase (AMPK), which measures the energy status of the cell, also stimulates the ULK complex (25). Two upstream kinases known as the tumor suppressor, liver kinase B1 (LKB1) and calcium/calmodulin-dependent protein kinase kinase-β (CaMKKβ), activate AMPK (26). The active AMPK phosphorylates ULK1 on multiple sites to stimulate the ULK1 complex and impedes mTORC1 via phosphorylating TSC2 and raptor (27). As a result, ULK1 is a point at which AMPK and mTOR regulate a certain metabolic pathway in opposing ways (28).

***Nucleation***

In autophagy, the process of mobilizing a small group of molecules toward the PAS is called nucleation. The nucleation process results in phagophore expansion through further recruitment of proteins (29). ULK1 protein phosphorylates and activates a class ІІІ phosphatidylinositol 3-kinase (PI3KC3) complex. This complex consists of Bcl-2-interacting myosin-like coiled-coil (Beclin1), Vacuolar protein sorting 34 (Vps34), Vps15, activation molecule in Beclin 1-regulated autophagy protein 1 (AMBRA1), UV radiation resistance-associated gene protein (UVRAG; also known as p63), and Atg14 (30). As a result of the activation of the PI3KC3 complex, PI3P is produced at the site of phagophore formation, which is essential for subsequent autophagy regulatory proteins to be recruited (31). By recruiting effector proteins, like WD repeat-domain phosphoinositide-interacting proteins (WIPIs) and zinc-finger FYVE domain-containing protein 1 (DFCP1), PI3P on the PAS prepares the phagophore for elongation (32).

***Elongation/expansion of phagophore***

The phagophore expands using multiple lipid sources to form enclosed vesicles called autophagosomes upon initiation and nucleation. Autophagosome formation is predominantly modulated by two ubiquitin-like conjugation systems, ATG5-ATG12 and ATG8-phosphatidylethanolamine (PE) (33). ATG12 binds ATG5 by E1-like ATG7 and E2-like ATG10, interacting with ATG16 to form the ATG12-ATG5-ATG16 complex. This complex promotes autophagosome membrane expansion (34). These proteins aid the ATG8 family members, which are comprised of the microtubule-associated protein 1A/1B-light chain 3 (LC3) and GABA type A receptor-associated protein (GABARAP) subfamilies, to bind the membrane lipids (35). LC3 is synthesized as pro-LC3 and then is processed and cleaved by ATG4 to produce a diffuse cytosolic form known as LC3-I. Subsequently, LC3-I conjugates to PE by Atg7 and Atg3 to form LC3-II (36). LC3-II can be located on the outer or inner side of the expanding phagophore’s membrane, and it plays an essential role in selecting substrates for lysis by the autolysosome. A mature phagophore eventually surrounds the destined cargo and fuses to form a double membrane phagosome (37). 

***Maturation and fusion***

Upon completion, autophagosomes undergo a maturation process that seems to occur almost simultaneously, along with separation from ER (38). Maturation is associated with the dissociation of PIP3 by phosphoinositide phosphatases and the cleavage of ATG8/LC3 through the action of the ATG4 protease family. As a result, a large portion of the autophagosome-associated ATG proteins dissociates from the complex, leading to the activation of the fusion machinery (39). Once most ATG machinery is released, the encapsulated cargo is transported into the acidic and hydrolytic lysosomal lumen to be degraded. This final step necessitates the fusion of the outer autophagosomal membrane with the lysosome, which is facilitated by several proteins, including soluble N-ethylmaleimide-sensitive factor attachment receptors (SNAREs), membrane-tethering complexes, and Rab GTPases (40). Rab proteins bind particular membranes and draw tethering complexes, which serve as a bridge to connect the compartments for fusion. Consequently, SNARE proteins use these tethering complexes to physically drive the fusion of opposing lipid bilayers (41).

***Targeting autophagy for breast cancer therapy***

Breast cancer is a heterogeneous tumor with distinct biological features that result in different response patterns to various therapies (42). Breast cancer can be divided into at least three subtypes based on the expression of estrogen receptor (ER), progesterone receptor (PR), and human epidermal growth factor receptor 2 (HER2). Triple-negative tumors (TNBC) lack ER, PR, and HER2 expression (43). Luminal breast cancer is classified into types A and B. Luminal A is the most common breast cancer subtype characterized by ER^+^ and/or PR^+^/HER2^−^ status (44). Luminal B accounts for 10% of all breast cancer cases and exhibits a lower expression of ER and low or no expression of PR. The HER2 subtype (HER2^+^/ER^−^/PR^−^) occurs in 20–25% of breast cancers worldwide (44). 

Autophagy plays a crucial role in breast tumor cell survival. This function of autophagy in improving/reducing breast tumor viability is primarily due to its antitumor and tumor-promoting properties (45). Besides proliferation, autophagy profoundly affects metastasis and invasion of breast tumors (45). Generally, autophagy promotes metastasis in the early stage, as it affects circulating tumor cells’ dissemination and survival and dormant tumor cells’ maintenance and survival. Autophagy, on the other hand, exhibits antimetastatic effects in the late stages of metastasis because it prevents the development of aggressive cancer cell subpopulations and macrometastatic growth. Due to autophagy’s complicated function, inhibiting or inducing autophagy in tumors requires careful consideration (10). 

Breast cancer molecular subtypes affect autophagy’s function differently and differ in the expression of autophagy-related proteins and metastatic organs (46). For instance, LC3-II overexpression is associated with a poor prognosis in TNBC, while it is not a biomarker in other breast tumor types. Autophagy can promote metastasis and decrease a patient’s response to therapy in TNBC (47). Additionally, autophagy-targeting treatments require biomarkers that accurately reflect autophagy status. For example, autophagy-related proteins such as Beclin1, LC3-I, LC3-II, and p62 indicate autophagy status in human breast tumors (48). 

Although manipulating autophagy for breast cancer therapy is fraught with challenges, modulating autophagy may be effective as a monotherapy or in combination with other treatments for breast cancer (49). Chemotherapy, radiation therapy, hormone therapy, and targeted therapy are currently available treatments for metastatic breast cancer (50). Stimulation of autophagy could enhance therapeutic effects by increasing the death of cancer cells. However, co-administration of an autophagy inhibitor during protective autophagy may alleviate treatment resistance and enhance therapeutic effects (10). The following sections discuss autophagy modulation in combination with conventional methods against breast cancer in preclinical and clinical studies. Table 1 shows the relationship between autophagy and different treatment methods for breast cancer.

***Radiation therapy and autophagy***

Radiation therapy is one of the three classical treatment modalities for breast cancer patients with lymph node metastases (51). However, it frequently fails to control tumor growth due to the development of resistance and dose-limiting side effects (51). Ionizing radiation (IR) is known to activate autophagy *in vitro* and *in vivo*, most likely by modulating endoplasmic stress and acting on the mTOR pathway. However, the underlying mechanism is unknown (52). 

Combining autophagy inhibitors and radiation therapy has demonstrated strong efficiency in treating cancer. For instance, autophagy inhibitors BafA1 and chloroquine (CQ) markedly boost radiation-induced death in MDA-MB 231 breast cancer cells (53). This increase in radio sensitivity seems to be due to the suppression of transforming growth factor-activated kinase 1 (TAK1) (53). Zhou *et al*. investigated the effects of inhibition of checkpoint kinase 1 (Chk1) on the radio-sensitivity of human TNBC cell lines MDA-MB-231, BT-549, and CAL-51 as well as MDA-MB-231 xenograft tumors in nude mice (54). According to their findings, irradiation significantly increased autophagy marked by enhancing the number of autophagosomes, elevating Atg5 levels, and stimulating the transformation of LC3-I to LC3-II. However, pretreatment with Chk1 inhibitor repressed the observed effects, suggesting that inhibition of Chk1 amplifies TNBC cell radio-sensitivity by obstructing irradiation-mediated autophagy (54). Another study revealed that the radio-resistant MDA-MB-231 breast cancer cells showed a noticeably higher degree of autophagy following IR than radiosensitive HBL-100 cells, indicating that autophagy serves a critical role in protecting breast cancer cells from IR (55). 

***Chemotherapy and autophagy***

By blocking autophagy, tumor cells may become more susceptible to standard medications or lose their chemotherapeutic resistance*.* CQ and its analog hydroxychloroquine (HCQ), as anti-malaria drugs, are the only recognized autophagy inhibitors that can be used for cancer therapy. CQ and HCQ improve the cytotoxicity of several medications, including 5-fluorouracil, cisplatin, and temozolomide (56). For instance, in a study, combination therapy using trastuzumab and CQ efficiently reduced breast tumor growth by >90% in HER2^+^ breast cancer xenografts resistant to trastuzumab (57). 

As the literature indicates, PI3K/AKT inhibitors can induce autophagy in different cancers, leading to drug resistance in aggressive tumors. In this regard, Cocco *et al*. have declared that two PI3K/AKT inhibitors, ipatasertib and taselisib, could induce autophagy in TNBC *in vitro* and *in vivo*. They showed that CQ, in combination with conventional paclitaxel (PTX), strongly potentiates the antitumor activity of PI3K/AKT suppressors (58). Researchers created a novel method based on co-delivery of an ATG7 siRNA and docetaxel (DTX) in a cross-linked, reducible, peptide-based micellar system for breast cancer treatment. Their research revealed that breast cancer cell lines vary significantly in their dependency on autophagy under normal or stressful circumstances. In addition, micellar siATG7 could successfully silence the ATG7 gene, hindered DTX-induced autophagy, and showed better anticancer effects (59). TNBC cell lines adapted to grow in the presence of 5-fluorouracil, doxorubicin, or docetaxel developed cross-resistance to chemotherapy and lower apoptotic sensitivity, as demonstrated by Das *et al*. Their further analyses used the autophagy inhibitor, BafA, and lentiviral knockdown of ATG5. Elevated cytoprotective autophagy was partially responsible for augmented cell survival and drug resistance in these cells (60). 

These results are inconsistent with some findings that point out the role of autophagy stimulation in improving the efficiency of conventional chemotherapy*.* In the microenvironment of breast tumors, adipocytes secrete many bioactive molecules known as adipocytokines. Among them, ADIPOQ/adiponectin is well known for its anticancer properties (61). Researchers found that ADIPOQ/adiponectin significantly slows the growth of breast cancer cells and triggers apoptosis through stimulation of autophagic flux. Accordingly, obstructing autophagosome formation, hindering autophagosome-lysosome fusion or genetic knockout Beclin1 and ATG7 effectively averts growth-inhibition and apoptosis stimulation caused by ADIPOQ/adiponectin. Moreover, they illustrated that co-treatment of ADIPOQ/adiponectin with various drugs, including carboplatin, PTX, or doxorubicin, raised the therapeutic efficacy of these chemotherapeutic medications (62). Bajbouj *et al*. discovered that despite the failure of estrogen therapy in inducing apoptosis, it improves the apoptotic effects of doxorubicin in MCF-7 cells. After assessing the expression of key autophagy and senescence markers as well as mitochondrial damage following estrogen therapy, they concluded that estrogen induces autophagy and a type of mitochondrial damage that results in cell senescence of breast cancer cells (63).

***Hormonal therapy and autophagy***

One of the most effective methods for treating ER^+^ breast cancer is using estrogen signaling regulators despite the occurrence of *de novo* and acquired resistance (64). Tamoxifen is a selective estrogen receptor modulator (SERM) that inhibits ER activity by competing with estrogen for this receptor, thereby lowering estrogen-mediated proliferation (65). Tamoxifen has been used as a hormonal medication for early and advanced ER^+^ breast cancer for over 40 years (65). However, primary or acquired resistance limits its effectiveness (66). Tamoxifen stimulates autophagy as a crucial mechanism for cell survival in tamoxifen-treated ER^+^ breast cancer cells (67). Thus, circumventing autophagy may provide a novel way to improve anti-estrogens’ effectiveness in combating tumors. According to a study, tamoxifen treatment of MCF7 cells raised the expression of lysosome-associated membrane protein 3 (LAMP3), a protein involved in autophagy. However, the shRNA-mediated knockdown of LAMP3 and/or the siRNA-mediated knockdown of autophagy-linked genes MAP1LC3B, ATG5, and BECN1 improved tamoxifen sensitivity (65). These findings have led to the development of numerous treatment strategies that target autophagy in combination with endocrine therapy. 

Thomas *et al.* reported that treating ER^+^ breast cancer cells with a combination of histone deacetylase (HDAC) inhibitor and tamoxifen triggered cell death. However, a subpopulation of cells showed resistance to apoptosis due to autophagy-associated survival caused by down-regulation of Bcl-2 and up-regulation of Beclin-1. Their results reveal that suppression of HDAC and autophagy resulted in the apoptosis of autophagy-protected cells and prevented tamoxifen resistance (64).

Aromatase inhibitors (AIs) such as the non-steroidal Anastrozole (Ana), Letrozole (Let), and steroidal Exemestane (Exe) are selective estrogen receptor down-regulators and considered the first-line hormone therapy in postmenopausal patients with breast cancer (68). Although the clinical efficacy of these medications for ER^+^ breast tumors has been proven, the main obstacle is the emergence of resistance (69). The role of autophagy as a pro-survival mechanism in Exe-induced cell death in ER^+^ sensitive breast cancer cells has been established (70). Researchers reported that Exe stimulates ER^+ ^breast cancer cells to undergo cell cycle arrest, apoptosis, and autophagy. According to their results, autophagy serves a pro-survival role by protecting breast cancer cells from apoptosis, thereby inducing Exe-acquired resistance (70). In a study, the role of autophagy in response to Ana and Let therapies and the effects of combining PI3K inhibitor BYL-719 with AIs were investigated in AI-resistant breast cancer cell lines. They described that Ana and Let treatments do not stimulate autophagy in resistant breast cancer cells (71).

Lapatinib is an FDA-approved HER2/EGFR inhibitor that represses the proliferation of metastatic HER2^+^ breast cancer (72). Researchers revealed anti- and pro-apoptotic functions of autophagy in breast cancer cells treated with lapatinib. They showed that short-term lapatinib therapy caused HER2^+^ breast cancer cells to undergo lapatinib-induced apoptosis reliant on autophagy, but extended lapatinib treatment led to the development of protective autophagy in the formerly established lapatinib-resistant cells. Moreover, it was discovered that inhibiting autophagy could revert lapatinib resistance in HER2^+^ breast cancer cells (73). 

Mele *et al*. pointed out that the inhibitory effect of lapatinib on breast cancer cells may be elevated by blocking glucose-6-phosphate dehydrogenase (G6PD), the limiting enzyme of the pentose phosphate pathway, through disrupting autophagy. Their data showed that the G6PD blockade prompted endoplasmic reticulum stress, contributing to the dysregulation of autophagy. Thus, G6PD inhibition directly enhances the lapatinib cytotoxic effect on breast cancer cells, while autophagy blocking reverses this effect (74). 

Trastuzumab, a monoclonal antibody specific for the extracellular region of HER2, is widely administrated as an approved first-line treatment for HER2^+^ breast cancers in combination with chemotherapy (75). Trastuzumab emtansine (T-DM1) is an approved drug for metastatic HER2^+^ breast cancer that comprises trastuzumab and microtubules inhibitor emtansine (DM1) (76). In a phase II clinical trial, the patients who received T-DM1 had improved progression-free survival compared to those receiving capecitabine and lapatinib treatment (77). Autophagy plays a significant role in the cytotoxicity effects of T-DM1 on HER2^+^ breast cancer cells. Blocking autophagy has diminished T-DM1-induced apoptosis in HER2^+^ breast cancer cells (76). This may propose the critical role of autophagy in T-DM1-induced cytotoxicity. Using quantitative proteomics, researchers identified that Atg9A is down-regulated in trastuzumab-resistant cells (BT474-TR) compared to trastuzumab-sensitive cells. They reported that Atg9A loss in trastuzumab-resistant cells enables HER2 to evade lysosomal-targeted degradation, indicating Atg9A as a target for anti-HER2 drugs to overcome therapy resistance (78). 

***Phytochemicals that target autophagy in breast cancer***

Plants produce phytochemicals or secondary metabolites to defend against environmental hazards such as UV radiation, fungus infection, and free radicals (79). Phytochemicals benefit the human body through various mechanisms, such as lowering inflammation or reactive oxygen species (ROS) (80). Phytochemicals are natural alternatives to chemopreventive agents for cancer therapy (81). In recent years, medicinal plants and their bioactive molecules have gained much attention to discover safe, effective, and affordable substances to overcome therapy resistance associated with conventional drugs or to avoid significant side effects of chemotherapeutics (82). Here, we review some of the most common phytochemicals that target the autophagy process to combat breast cancer (Table 2). The chemical structure of these phytochemical compounds is shown in Figure 2.

***Artemisinin***

Artemisinin is an active compound isolated from the medicinal plant *Artemisia annual*. Its water-soluble semisynthetic derivative, Artesunate, has widely been used to cure mild to severe malaria worldwide (31). Accumulating evidence has unraveled the potential anticancer effect of these two compounds by acting on autophagy (83). In MCF-7 and MDA-MB-231 breast cancer cells, artesunate was capable of inhibiting the proliferation of these cancer cells through inducing autophagy, which was indicated by up-regulation of Beclin-1 and aggregation of LC3-II and increasing sensitivity to the chemotherapy drug epirubicin (84). Guan *et al*. found that artemisinin had strong anticancer effects on cisplatin-resistant MDA-MB-231 cells by inducing apoptosis, autophagy, and cell cycle arrest. Artemisinin promoted the production of autophagosomes and autophagic vacuoles to stimulate autophagy in these breast cancer cells (85). A study indicated that artemisinin inhibited autophagy in the MCF-7 cell line. The inhibitory activity of Artemisinin in this cell line was exerted by hindering autophagosome turnover and inducing perinuclear clustering of autophagosomes, early and late endosomes, and lysosomes (86). A targeted liposome containing artesunate was developed by researchers to enter breast cancer cells and affect mitochondria *in vitro* and *in vivo* (87). This liposome prevented the proliferation of tumor cells by inducing mitophagy in MDA-MB-231 cells and xenograft tumors, characterized by decreased PHB2 (mitophagy receptor) expression and increased LC3-II and PINK1 (a mitochondrial gatekeeper) expression.

***Baicalein***

Baicalein is a commonly used Chinese herbal medication isolated from *Scutellaria* *baicalensis* with anticancer effects on numerous human cancer cell lines (88, 89). The *in vitro* and *in vivo* treatment of breast cancer cells with baicalein exhibited the potential of this compound in suppressing cell proliferation, triggering apoptosis, and stimulating autophagy. In MCF-7 and MDA-MB-231 cells, baicalein regulated apoptosis and autophagy through the PI3K/AKT pathway. Baicalein-treated cells formed autophagic vacuoles and showed significantly increased levels of LC3II and BECN1 in a dose- and time-dependent manner. There was also a dose- and time-dependent reduction in p-AKT/AKT and p-mTOR/mTOR ratios. The results of *in vitro* experiments were consistent with data obtained using xenograft nude mice (90). Baicalein has been shown to improve the doxorubicin chemosensitivity of TNBC cells. In MDA-MB-231 cells, baicalein prevented doxorubicin-induced decrease in autophagy markers and up-regulated CDK1 expression (91). Aryal *et al*. demonstrated that baicalein could induce autophagic cell death in breast cancer cells. They discovered that baicalein induced human breast cancer cell death by inducing autophagy rather than apoptosis. It significantly increased the expression levels of LC3B-II, autophagosome formation, and autophagic flux. Baicalein-induced autophagy was found to be mediated by acting on Beclin 1, Vps34, Atg5, Atg7, and ULK1. Furthermore, this compound activated ULK1 by acting on AMP-activated protein kinase (AMPK)α and blocking mTOR and Raptor, upstream inhibitors of ULK1 and autophagy (92). 

***Britannin***

Britannin is a sesquiterpene lactone, a diverse group of phytochemicals with many biological properties such as pro-apoptotic activity (93). Rajabi *et al*. evaluated the effects of britannin on apoptosis and autophagy in MCF-7 breast cancer cells. Their data uncovered that this sesquiterpene could significantly induce apoptotic cell death in MCF-7 cells by up-regulating caspase-3 through a mechanism that involved the blockage of the JAK/STAT pathway. Interestingly, britannin treatment down-regulated the levels of four autophagy markers, including ATG4, ATG5, Beclin1, and LCIII in MCF-7 cells. Consequently, they suggested britannin as a drug candidate for the suppression of breast cancer (94).

***Celastrol***

Celastrol, a tripterine derived from the *Tripterygium wilfordii *Hook F (TWHF), is one of the most promising natural medicine compounds. As indicated by encouraging outcomes of multiple preclinical studies, celastrol has anticancer activities against different tumors, such as breast cancer (95). Researchers designed a celastrol derivative with a strong antiproliferative property against MCF-7 cells. Regarding the elevated expression of LC3 A/B, p62, and Beclin-1 in these cancer cells, they suggested that the significant antiproliferative activity of this compound was primarily due to its ability to induce autophagy (96). Another study has declared that celastrol and tamoxifen have synergistic anticancer effects by causing MCF-7 cells to undergo apoptosis and autophagy. The combination treatment triggered autophagy by reducing p-Akt and p-mTOR levels in MCF-7 cells and enhanced the autophagy marker proteins such as LC3II and P62 (97).

***Cucurbitacin B***

Cucurbitacin B (Cuc B) is a naturally occurring tetracyclic triterpene phytochemical in various plant species, especially in the *Cucurbitaceae* family (98). Documented results have proven the antiproliferative impacts of Cuc B on a series of cancer cell lines, including breast cancer cell lines both *in vitro* and *in vivo* (99-101). A study explained that Cuc B significantly hindered the viability of MCF-7 cells via ROS-mediated DNA damage and autophagy as verified by overexpression of autophagic proteins LC3-II, Beclin-1, p-ULK1, and monodansylcadaverine (MDC) staining, an autofluorescent probe to identify the autophagic vesicles. In addition, the treatment with Cuc B inhibited the expression of p-mTOR, p-Akt, and p62 proteins (102). 

***Curcumin***

Curcumin, also known as diferuloylmethane, is a natural and biologically active polyphenol derived from the rhizome of the *Curcuma longa* (turmeric) plant with therapeutic applications for centuries (103). Availability and relative safety make curcumin a promising option for cancer therapy (104). Xiao-ai *et al*. demonstrated curcumin induced chemo-sensitivity in multidrug-resistant (MDR) breast cancer cells. Their data from *in vivo* and *in vitro* experiments suggested that curcumin may trigger autophagy to sensitize MDR breast cancer cells to the chemotherapy drug cisplatin. This process was mediated by the down-regulation of long noncoding RNA Colon-cancer-associated transcript-1 (CCAT1) and inactivation of the PI3K/Akt/mTOR pathway in treated breast cancer cells. Moreover, in xenograft nude mice models of MCF-7/DDP breast cancer, curcumin was successfully used to resensitize resistant breast cancer tumors to cisplatin (105). According to recent data, irradiation and curcumin nanoparticles could induce autophagy, apoptosis, and G0/G1 cell cycle arrest in MD-MB-231 breast cancer cells. Autophagic vacuoles were observed in treated cell lines after increased curcumin concentration (106). In another study, co-treatment of curcumin and berberine induced apoptotic and autophagic cell death in MCF-7 and MDA-MB-231 cells. In contrast to treating breast cancer cells with either agent separately, co-treatment of these two compounds induced autophagy more potently, as demonstrated by the formation of autophagic vacuoles, the turnover of LC3-II, and the degradation of p62 (107).

***Gaillardin***

Gaillardin is a novel member of the sesquiterpene lactones with significant anti-cancer activities. An *in vitro* study was conducted to assess the effects of gaillardin on apoptotic and autophagy markers in the MCF-7 breast cancer cell line. The obtained data unraveled that gaillardin suppressed the proliferation of these cancer cells by inducing apoptosis and inhibiting autophagy. Gaillardin elevated the expression of the activated form of the caspase-3 enzyme to induce apoptosis in MCF-7 cells. Moreover, it hampered the expression of six autophagy markers, including ATG1, ATG4, ATG5, ATG12, Beclin1, and LC-III in these breast cancer cells. Conclusively, the authors of that study suggested gaillardin as a novel agent for the treatment of breast cancer (108).

***Icariin***

Icariin is a natural flavonoid isolated from *Epimedium* *brevicornum* *Maxim* with antitumor properties (109-111). According to a study, icariin treatment of tamoxifen-resistant MCF-7 cells not only hampered their proliferation but also reversed the resistance to tamoxifen in these cells. This was achieved by suppressing autophagy, resulting in cell cycle arrest and apoptosis stimulation. Icariin inhibited autophagy by reducing LC3-1, LC3-II, AGT5, and Beclin-1 expression levels, along with enhancing p62. The important role of autophagy was supported by evidence showing that autophagy inhibition via 3-MA meaningfully boosted the effects of icariin on cell viability and apoptosis (112). A study uncovered that IC2, a derivative of icariin, potentially inhibits breast cancer cell growth. IC2 inhibited stearoyl-CoA desaturase 1 (SCD1) to promote cytoprotective autophagy in MCF-7 and MDA-MB-231 cells. IC2 stimulated autophagy in these breast cancer cell lines via the AMPK/mTOR pathway by phosphorylation of AMPK and inhibition of mTOR. Additionally, their *in vivo* experiments revealed that IC2 treatment inhibited tumor growth in a breast cancer xenograft model (113).

***Paclitaxel***

Paclitaxel (PTX) is a member of taxanes and a common chemotherapeutic medication used to treat breast cancer (114). Acting as microtubule-stabilizing agents, taxenes affect autophagy, since microtubules contribute to the pre-autophagosomal structures, autophagosome movement toward the lysosome as well as the regulation of two key complexes that trigger autophagic response, including mTORC1 and PI3K-III (77). Some data suggest that increased levels of key autophagy regulator Beclin1 protect tumor cells from the cytotoxic effects of PTX on various cancers, such as ovarian cancer, non-small cell lung cancer (NSCLC), and nasopharyngeal carcinoma (115-117). In BT474 breast cancer cells, PTX inhibits cell viability and Beclin1 expression levels. In addition, PTX significantly reduces tumor growth in a BT474 xenograft model with Beclin1 knockdown compared to the control group, implying that Beclin1 prevents breast cancer cell apoptosis (114). The anticancer effectiveness of PTX is significantly diminished due to the development of resistance in cancer cells. A major cause of resistance is PTX-induced cytoprotective autophagy, which occurs by various mechanisms depending on the cell type and may even lead to metastases. Cancer cells are resensitized to PTX by autophagy inhibitors (118). Wang *et al*. found that PTX-resistant TNBC cells showed enhanced starvation-induced autophagy and displayed a significant reduction in cell viability, growth, and invasion upon being treated with autophagy inhibitors or silencing autophagy regulator of eukaryotic elongation factor 2 kinase (eEF2K). These findings documented the critical part of autophagy in chemoresistance and aggressive behavior of TNBC (119). Moreover, pharmacological inhibition of autophagy by spautin-1 or knockdown of FIP200 and Atg13 genes in PTX-resistant MDA-MB-231 cells resulted in an accumulation of damaged mitochondria. The PTX-resistant cells were more likely to die when autophagy was inhibited (120). A study provided evidence to highlight that tumor necrosis factor-α-induced protein-8-like-2 (TIPE2) suppressed drug-induced autophagy, which sensitized breast cancer cells to PTX. In MCF-7 and MDA-MB-231 cells, TIPE2 inhibited PTX-induced autophagy by activating AKT/mTOR and inhibiting TAK1/MAPK signaling pathways. Breast cancer cells treated with PTX showed increased expression of the autophagy-associated proteins Beclin1 and LC3B (LC3II/I), but these proteins were down-regulated when TIPE2 was overexpressed (121).

***Resveratrol***

Resveratrol, a non-flavonoid polyphenol found in many foods like grapes, berries, soybeans, pomegranate, and peanuts, has been proven to have various positive health effects. For instance, the antitumor effects of resveratrol have been reported in several breast cancer cell lines (81). Resveratrol makes breast cancer cells more susceptible to a poly ADP-ribose polymerase inhibitor (PARPi), known as talazoparib, by simultaneously suppressing AKT signaling and autophagy flux. Resveratrol acts on autophagy cell death by inhibiting autophagosome and lysosome fusion through increasing lysosomal-membrane-permeabilization (122). Researchers evidenced the effects of a combination of therapeutics using the allosteric mTORC1 inhibitor of rapamycin with an autophagy inhibitor resveratrol. Their results suggested that the combination of two drugs upholds mTORC1 suppression while impeding Akt activation and autophagy increase, which resulted in apoptosis of ER^+^ (MCF7) and ER^-^ (MDA-MB-231) breast cancer cells (123). Aza resveratrol analogs have been revealed to initiate cell death in MDA-MB-231 and T47D breast cancer cells through autophagy induction, as shown by Beclin1 up-regulation (124). A study conducted found that resveratrol inhibited breast cancer stem cells and induced autophagy by suppressing the Wnt/b-catenin pathway. A significant increase in autophagy markers, LC3-II, Beclin1, and Atg 7, was observed in that study (125). Wang *et al*. reported that resveratrol inhibited invasion and metastatic spread of breast cancer cells by reversing epithelial-mesenchymal transition (EMT), which was mediated by transforming growth factor-beta 1 (TGF-β1) stimulatory effect. Resveratrol’s anticancer activity was mediated by autophagy in 4T1 breast cancer cells and xenograft mice, as resveratrol increased autophagy-related gene and protein levels. Resveratrol stimulated autophagy by phosphorylating AMPK and enhancing the expression of SIRT3 in breast cancer cells (126). According to a study, resveratrol synergistically enhanced the anticancer activity of salinomycin against TNBC (MDA-MB-231) and a mice model. The combination treatment of resveratrol and salinomycin decreased the expression of Beclin, LC3-I, and LC-II in MDA-MB-231 cells. Further, this combination acted synergistically against cancer cell proliferation by inducing apoptosis via raising Bax/Bcl-2 ratios (127). Scarlatti *et al*. proposed that resveratrol activated both caspase-dependent and independent cell death in MCF-7 cells concomitantly to stimulate Beclin 1-independent autophagy. They suggested that this non-canonical autophagy, induced by resveratrol, may serve as a caspase-independent cell death mechanism in breast cancer cells (128).

***Tetrandrine***

Tetrandrine, a natural bisbenzylisoquinoline alkaloid extracted from the root tuber of *Stephania tetrandra*, was proven to have potential antitumor activities (129). The promising results were revealed following the treatment of TNBC MDA-MB-231 cells with tetrandrine, as it blocked the proliferation and stimulated apoptosis of these cancer cells. According to research, tetrandrine prompted MDA-MB-231 cell autophagy by reducing p62/SQSTM1 expression, elevating the expression of Beclin1 and LC3-II/LC3-I as well as suppressing the PI3K/AKT /mTOR pathway (130). A study presented evidence showing that tetrandrine amplified autophagic flux in breast cancer cells, leading to cell death. The same outcome was observed when apoptosis-resistant cell lines were exposed to tetrandrine. It was also detected that tetrandrine initiates mTOR-dependent autophagy via direct inhibition of protein kinase C–α (PKC-α) (131). Wang *et al*. clarified that tetrandrine could resume the sensitivity of tamoxifen-resistant breast cancer cells to tamoxifen. Tetrandrine significantly suppressed the proliferation of tamoxifen-resistant cells. This was attributed to the tetrandrine-induced proapoptotic effect of tamoxifen via inhibition of autophagy. Their claim was affirmed by observing augmented levels of autophagosomes, LC3-II, and p62 in tamoxifen-resistant cells after being treated with the combination of tetrandrine and tamoxifen (132). A study found that tetrandrine enhanced arsenite cytotoxicity in MCF-7 cells synergistically. The combination of tetrandrine and arsenite exerted an autophagy-inducing effect on MCF-7 cells, and thereby, several autophagy-related proteins such as LC3-I, LC3-II, ATG7, Beclin-1, and AMPK were up-regulated while mTOR was down-regulated (133).

***Thymoquinone***

Thymoquinone is the principal phenolic constituent of the oil of black seeds of *Nigella sativa*. Thymoquinone exhibits promising pharmacological effects and acts on the signaling pathways that promote cancer growth. It increases the anticancer potency of chemotherapy medications and alleviates their adverse effects (134). Thymoquinone has been reported to suppress cell proliferation and TNBC migration by inhibiting autophagy and hampering the expression of Beclin-1 and LC3 (135). An investigation showed that thymoquinone synergizes gemcitabine’s anti-breast cancer effects by modifying its apoptotic and autophagic capacities (136). A combination of thymoquinone and PTX induced autophagy in MCF-7 and T47D cells more significantly than the two agents alone. Interestingly, this combination only significantly increased Beclin-1 and LC3-II expression in MCF-7 (137). The combination of docetaxel and thymoquinone in a nanocarrier had a synergistic effect on apoptosis in breast cancer cells, which was linked with the induction of autophagy. Compared to untreated cells, this combined treatment significantly increased autophagic vesicles in MCF-7 and MDA-MB-231 cells (138). 

***Tocotrienols***

Tocotrienols are members of the vitamin E family and isoprenoid compounds found in various vegetable oils, wheat germ, barley, and some types of nuts and cereals (139). Previous research has shown that tocotrienols have potent anticancer activity with negligible or no adverse effects on the function or viability of normal cells (140). Tiwari *et al*. pointed out that treatment of mouse and human mammary tumor cell lines with γ-tocotrienol diminished the viability of these cancer cells with a concomitant rise in autophagic and ER stress indexes. Besides, blockage of autophagy with some inhibitors such as Beclin-1 siRNA, 3-methyladenine (3-MA), or bafilomycin A1 (Baf1) decreased γ-tocotrienol-induced cytotoxicity (141, 142). According to another study by the same group, the combination of natural phytochemical oridonin and γ-Tocotrienol induced synergistic autophagic and apoptotic effects on malignant +SA mouse mammary epithelial cells with no impact on normal cells. The expression of autophagy markers, including Beclin-1, Atg3, Atg7, Atg5-Atg12, LAMP-1, and cathepsin-D, and the conversion of LC3B-I to LC3B-II, was considerably increased by this combination while exposure to autophagy inhibitors 3-MA or Baf1 reversed all these effects (143).

***Ursolic acid***

Ursolic acid is a triterpenoid phytochemical broadly distributed in medicinal herbs, fruits, and vegetables. Terpenoids are promising candidates as chemopreventive agents for cancer treatment. Anti-inflammatory, anti-proliferative, and pro-apoptotic actions of terpenoids, both *in vitro* and *in vivo,* are the basis for their anticancer activity. Ursolic acid is capable of inducing apoptosis in phenotypically distinct MCF-7 (ER^+^, PR^+/−^, HER2^−^), MDA-MB-231 (ER^−^, PR^−^, HER2^−^), and SK-BR-3 (ER^−^, PR^−^, HER2^+^) breast cancer cells via targeting the glycolytic pathway and autophagy (144). This terpenoid has been reported to sensitize MCF-7 and MDA-MB-231 cells to epirubicin (EPI) by acting on the autophagy pathway in these cells. Wang *et al*. discerned that the concurrent use of ursolic acid and EPI raises the expression of autophagy-related proteins Beclin-1, LC3-II/LC3-I, Atg5, and Atg7, while down-regulating PI3K and AKT levels, which is likely achieved by controlling class III PI3K(VPS34)/Beclin-1 pathway and PI3K/AKT/mTOR pathway. Furthermore, autophagy inhibitor 3-MA could reverse observed effects on these proteins (145). According to the findings of a study, ursolic acid reduced breast cancer cells’ ability to proliferate via activating autophagy and apoptosis. After ursolic acid treatment, all cell lines T47D, MCF7, and MDAMB231 exhibited markedly reduced expression of PI3K and diminished levels of phosphorylated AKT. Ursolic acid, however, significantly up-regulated the levels of LC3 mRNA in three cell lines (146). Ursolic acid triggered ER stress and autophagy in MCF-7 breast cancer cells. However, autophagy-dependent ER stress protected the cancer cells from ursolic acid-induced apoptosis by up-regulating MCL1 via EIF2AK3. It was suggested that MAPK1/3 activation was the main cause of ursolic acid-induced cytoprotective autophagy. The cytoprotective effect of ursolic acid was only observed at low concentrations, but higher concentrations led to apoptosis induction (147).

**Concluding remarks and future perspectives**

Autophagy is a highly conserved catabolic process that degrades cytoplasmic proteins and organelles in the lysosomes. Thus, it is a physiological process necessary for normal tissue homeostasis. However, our knowledge of the tangled relationship between autophagy and cancer is still unclear. According to studies, autophagy is intricately linked to numerous biological processes, including metabolism, stress response, and programmed cell death (148-150). The prevailing opinion is that autophagy serves a dual role in tumor promotion and suppression. Although inducing autophagy may help prevent cancer by restricting chronic inflammation and tumor necrosis, inhibiting autophagy-mediated tumor cell survival is more likely to be effective in cancer therapy (151). As described before, current antineoplastic regimens for breast cancer, including radiotherapy, chemotherapy, endocrine therapy, and targeted therapy, have been established to stimulate autophagy as a pro-survival process. Hence, it seems reasonable to expect that the therapeutic efficacy of these treatment modalities can be improved if autophagy is blocked. Among numerous agents that target autophagy, FDA-approved antimalarial compounds are being applied in several cancer clinical trials as autophagy inhibitors. For instance, CQ and HCQ, as well as artemisinin and artesunate, can directly modulate autophagy, thereby potentiating available classical methods of cancer therapy, especially chemotherapy (152, 153). Therefore, these compounds are used as adjuvant therapies for the treatment of breast cancer patients. However, possible long-term side effects on normal cells and the functions of key organs are crucial factors that need to be weighed in the clinical applications of autophagy inhibitors. In this regard, plant-derived products can be considered promising adjuvants for conventional therapeutics for cancer treatment, especially to overcome drug resistance. Besides, they could be the practical alternatives to induce cell death in apoptosis-resistant cells (154). 

We reviewed the most common phytochemicals that can control autophagy in breast cancers. According to the literature, phytochemicals may stimulate or suppress autophagy and may inhibit or trigger apoptosis. Additionally, our literature review shows that some phytochemicals can induce cytotoxic autophagy, which allows for an alternative way for cell death in apoptosis-resistant cells. Although employing phytochemicals has beneficial effects such as low toxicity, which make them suitable candidates for single or combination cancer therapy, some clinical issues remain unsolved. First, poor bioavailability of these phytochemicals is an obstacle that may restrict their application. Therefore, further studies on drug delivery systems based on nanotechnology can be used to combat this issue. Second, developing anticancer medicines based on phytochemicals is further challenging because some natural compounds target several signaling pathways that may be shared among various biological systems. It is the same issue that diminishes the effectiveness of chemotherapeutics since it causes toxicity to both normal and cancerous cells. Studies on phytochemical-mediated autophagy conducted *in vitro* and *in vivo* may solve this problem. The last issue is not only employing phytochemicals but also targeting autophagy as an option for cancer treatment. Since autophagy has a context-dependent role in cancers, its targeting may not always be advantageous. Even within the various subtypes of breast cancer, autophagy may play conflicting roles; for instance, it inhibits tumor initiation and anchorage-independent growth in TNBC (155). As a result, the stage of the disease and its autophagic characteristics must also be considered when discussing the therapeutic role of autophagy. It is crucial to shed more light on the role of autophagy in breast cancer, particularly regarding possible prognostic and predictive autophagy markers to provide a therapeutic benefit for patients. Accordingly, finding and confirming biomarkers that track autophagy status *in vivo* and applying effective phytochemicals as autophagy modulators may enable researchers to forecast early versus late autophagy modulation outcomes for breast cancer treatment. 
